# Impact of dose and administration schedule on biologic efficacy in chronic rhinosinusitis with nasal polyps: a network meta-analysis

**DOI:** 10.3389/fphar.2026.1772373

**Published:** 2026-08-07

**Authors:** Mostafa A. Khalifa, Aya Ahmed Shimal, Mohamed H. Mahmoud, Bakri Roumi Jamal, Peter Atef, Mohamed Sherif Ali Ahmed, Ibrahim Khalil, Monsurah Bisola Alatise, Ali Dway, Sarah Raied Irhayyif

**Affiliations:** 1 Faculty of Medicine, Cairo University, Cairo, Egypt; 2 College of Medicine, University of Baghdad, Baghdad, Iraq; 3 Faculty of Medicine, Alexandria University, Alexandria, Egypt; 4 Faculty of Medicine, University of Aleppo, Aleppo, Syria; 5 Alexandria Faculty of Medicine, Alexandria, Egypt; 6 Mansoura Faculty of Medicine, Mansoura, Egypt; 7 Dhaka Medical College and Hospital, Dhaka, Bangladesh; 8 Faculty of Medicine, Cadi Ayyad University, Marrakech, Morocco; 9 Faculty of Medicine, Al-Andalus University for Medical Sciences, Tartus, Syria

**Keywords:** benralizumab, biological products, chronic rhinosinusitis, CRSwNP, dupilumab, mepolizumab, nasal polyps, omalizumab

## Abstract

**Objective:**

Chronic rhinosinusitis with nasal polyps (CRSwNP) severely impairs quality of life despite standard surgical and medical therapies. We conducted a network meta-analysis to compare the relative efficacy of biologic agents targeting type 2 inflammation, with a specific focus on how dose and administration frequency influence patient-reported and objective outcomes.

**Methodology/Principal:**

A frequentist network meta-analysis was performed across randomized controlled trials evaluating dupilumab, omalizumab, mepolizumab, and benralizumab versus placebo in adults with CRSwNP. Primary outcomes evaluated included sinonasal quality of life (Sino-Nasal Outcome Test-22 [SNOT-22]), objective polyp burden (Nasal Polyp Score [NPS]), and olfactory function (University of Pennsylvania Smell Identification Test [UPSIT]). Interventions were ranked using P-scores.

**Results:**

Sixteen randomized controlled trials (4,861 patients) were included. Dupilumab (600 mg loading dose followed by 300 mg every 1–2 weeks) ranked as the most effective regimen for improving quality of life (P-score = 0.907) and provided the greatest enhancement in olfactory function (P-score = 0.006; Standardized Mean Difference [SMD] = 1.91, 95% confidence interval [CI]: 1.24–2.59). Conversely, benralizumab 30 mg administered every 4 weeks was the top-ranked intervention for reducing objective polyp burden (P-score = 0.864). Substantial heterogeneity and significant global inconsistency were observed within the SNOT-22 ( $I^{2}=86.5\%$ ) and NPS ( $\left.I^{2}=68.9\%\right)$ networks, whereas the UPSIT network demonstrated minimal heterogeneity $(I^{2}=7.5\%$ ) and remained statistically consistent.

**Conclusion:**

Biologic efficacy in CRSwNP varies distinctly by clinical endpoint and dosing frequency. Dupilumab optimally drives patient-reported quality of life and olfactory recovery, while intensive benralizumab dosing (every 4 weeks) provides the greatest objective reduction in nasal polyp burden. Therapeutic selection should be personalized to align with individual patient phenotypes and clinical priorities.

## Introduction

The 2020 European Position Paper on Rhinosinusitis and Nasal Polyps defines chronic rhinosinusitis with nasal polyps (CRSwNP) as the presence of two or more symptoms, one of which must be either nasal blockage/obstruction/congestion or nasal discharge (anterior or posterior nasal drip), with or without facial pain/pressure, and/or a reduction or loss of smell lasting for more than 12 weeks. This must be accompanied by endoscopic confirmation of nasal polyps in the middle meatus (Fokkens et al., 2020). CRSwNP affects approximately 2% to 4% of adults (Sedaghat et al., 2022; Chen et al., 2020). Patients with comorbid asthma or nonsteroidal anti-inflammatory drug (NSAID)-exacerbated respiratory disease tend to experience more severe disease, frequent recurrence of nasal polyps after surgery, higher systemic corticosteroid use, and poor asthma control. These factors negatively impact quality of life, including work productivity, sleep, physical activity, and are associated with increased healthcare costs and utilization (Sedaghat et al., 2022; Rank et al., 2023).

The current standard of care includes saline nasal irrigation, intranasal corticosteroids, short courses of systemic corticosteroids, and endoscopic sinus surgery to remove polyp tissue and diseased mucosa in severe cases (Fokkens et al., 2020; Chen et al., 2020; Rank et al., 2023). However, recurrence after surgery is common, and the procedure carries risks. Recently, other treatment options have emerged, including aspirin desensitization and biologic therapies. Biologics such as dupilumab (an IL-4 and IL-13 signaling inhibitor), omalizumab (an anti-IgE monoclonal antibody), and mepolizumab (a monoclonal antibody targeting IL-5) are designed to target the type 2 inflammatory pathways that underlie the pathogenesis of CRSwNP (Bachert et al., 2019; Gevaert et al., 2024; ; Wang et al., 2025).

Despite the increasing availability of biologics, few studies have directly compared their efficacy and safety. Previous systematic reviews and network meta-analyses primarily focused on comparing biologic agents as a class, without evaluating how *dose and frequency of administration* influence treatment outcomes. Optimizing both the choice of biologic and its dosing regimen is highly relevant to clinical decision-making, yet has been overlooked (Wang et al., 2025). To address this gap, we conducted a network meta-analysis (NMA) to evaluate and compare the efficacy of all currently available biologics for the treatment of CRSwNP, with a particular focus on differences in dosing regimens and administration frequencies. Key outcomes included reduction in Nasal Polyp Score (NPS), improvement in nasal congestion and sense of smell, enhancement of quality of life as measured by the SNOT-22 questionnaire, and a reduction in the need for systemic corticosteroids or surgical intervention.

## Methods

This network meta-analysis (NMA) was conducted and reported in accordance with the Preferred Reporting Items for Systematic Reviews and Meta-Analyses extension for Network Meta-Analyses (PRISMA-NMA) guidelines (Hutton et al., 2015).

### Eligibility criteria

This study included randomized controlled trials (RCTs), and observational cohort studies (prospective or retrospective) that evaluated the efficacy of monoclonal antibody therapies, such as Dupilumab, Omalizumab, Mepolizumab, and Benralizumab, administered at any dose or frequency, compared to placebo or other monoclonal antibody therapies, in adult patients (≥18 years) diagnosed with chronic rhinosinusitis with nasal polyposis (CRSwNP), confirmed by clinical guidelines such as the European Position Paper on Rhinosinusitis and Nasal Polyps (Fokkens et al., 2020). The primary outcomes assessed were the Sino-Nasal Outcome Test-22 (SNOT-22) score, Nasal Polyp Score (NPS), and University of Pennsylvania Smell Identification Test (UPSIT) score, reported as continuous variables. Studies were excluded if they involved pediatric populations, conditions other than CRSwNP (e.g., allergic rhinitis without polyposis), non-monoclonal antibody interventions, or lacked a comparator arm.

### Information sources and search strategy

We searched PubMed, Embase, Cochrane Central Register of Controlled Trials (CENTRAL), and Web of Science from inception to February 2026. Clinical trial registries (ClinicalTrials.gov) were also searched for unpublished trials. The search strategy combined Medical Subject Headings (MeSH) and free-text terms for CRSwNP (e.g., “chronic rhinosinusitis,” “nasal polyps”), monoclonal antibodies (e.g., “Dupilumab,” “Omalizumab,” “Mepolizumab,” “Benralizumab”), and study designs (e.g., “randomized controlled trial,” “cohort study”).

### Study selection

Two reviewers independently screened titles and abstracts using Rayyan software, followed by full-text review. Discrepancies were resolved through discussion or by a third reviewer.

### Data collection process

Data were extracted independently by two reviewers using a standardized Microsoft Excel form. Extracted items included study characteristics (author, year, design, sample size), population details (age, sex, CRSwNP diagnostic criteria), intervention details (drug, dose, frequency, duration), comparator details, and outcome data (SNOT-22, NPS, UPSIT scores, including means and standard deviations). Corresponding authors were contacted for missing data. Discrepancies were resolved through consensus.

### Quality assessment

Two independent reviewers conducted a risk-of-bias assessment for the included studies. For the included randomized controlled trials (RCTs), the Cochrane Risk of Bias 2 (RoB 2) tool was used to evaluate domains including the randomization process, deviations from intended interventions, missing outcome data, measurement of outcomes, and selection of reported results. Each domain was assessed and categorized as low risk of bias, some concerns, or high risk of bias according to the RoB 2 guidance (Sterne et al., 2019).

### Data synthesis and statistical analysis

The statistical analysis was performed using the netmeta package in R software, with a network meta-analysis based on a frequentist effects model used to evaluate the efficacy of various treatment options across three continuous variables: SNOT-22, NPS, and UPSIT. Treatments were analyzed according to specific biologic dose and administration frequency to allow comparison of different dosing regimens. Treatment arms were grouped into nodes based on similarity in dosing regimen and availability of outcome-specific data. For benralizumab, dosing schedules (e.g., every 4 weeks, every 8 weeks with or without loading doses) were considered separately or combined depending on how they were reported within each outcome network. As a result, the number and definition of nodes varied across outcomes. Single-dose regimens were included when outcome-specific data were available and could be appropriately connected within the network structure. This approach enables a more detailed evaluation of dosing strategies but may reduce the amount of evidence contributing to individual comparison nodes, potentially increasing uncertainty around some estimates. The validity of indirect comparisons in network meta-analysis relies on the assumption of transitivity, meaning that the included studies are sufficiently comparable in terms of clinical and methodological characteristics that may influence treatment effects. In this study, we evaluated the similarity of trials regarding patient characteristics, disease severity, intervention regimens, and outcome definitions across studies. Consistency between direct and indirect evidence was assessed using node-splitting analysis. To account for potential variations in measurement methods across studies, standardized mean differences (SMD) were used to pool effect sizes. SMDs were reported with 95% confidence intervals (CI), where intervals crossing 0 indicated no statistically significant difference between interventions. To enhance clinical interpretability, pooled standardized mean differences (SMDs) were also expressed as approximate changes in the original outcome scales (SNOT-22, Nasal Polyp Score [NPS], and University of Pennsylvania Smell Identification Test [UPSIT]) using representative pooled standard deviations derived from the included trials. These estimates were interpreted in relation to established minimal clinically important differences (MCIDs) where available. Heterogeneity was assessed using the chi-squared test, with P < 0.1 and I^2^ ≥ 50% thresholds indicating substantial heterogeneity. Homogeneous data were analyzed using a fixed effects model, while a random effects model was applied for heterogeneous data. Consistency within the network was evaluated through comparisons of direct and indirect treatment effects. The node-splitting approach was utilized, with a P-value threshold of P < 0.05 indicating significant inconsistency. Additionally, the global inconsistency test based on the design-by-treatment interaction model was conducted, with the same significance threshold (P < 0.05) applied to identify inconsistency in the overall network. Intervention effectiveness for each outcome was ranked using the P-score method, which provides a scale ranging from 0% (least effective) to 100% (most effective). To ensure robustness of the findings, sensitivity analyses were performed by excluding studies assessed as having a high risk of bias, reinforcing the reliability of the network estimates. Publication bias was examined using funnel plots and Egger’s regression test.

## Results

### Study selection

**FIGURE 1:**
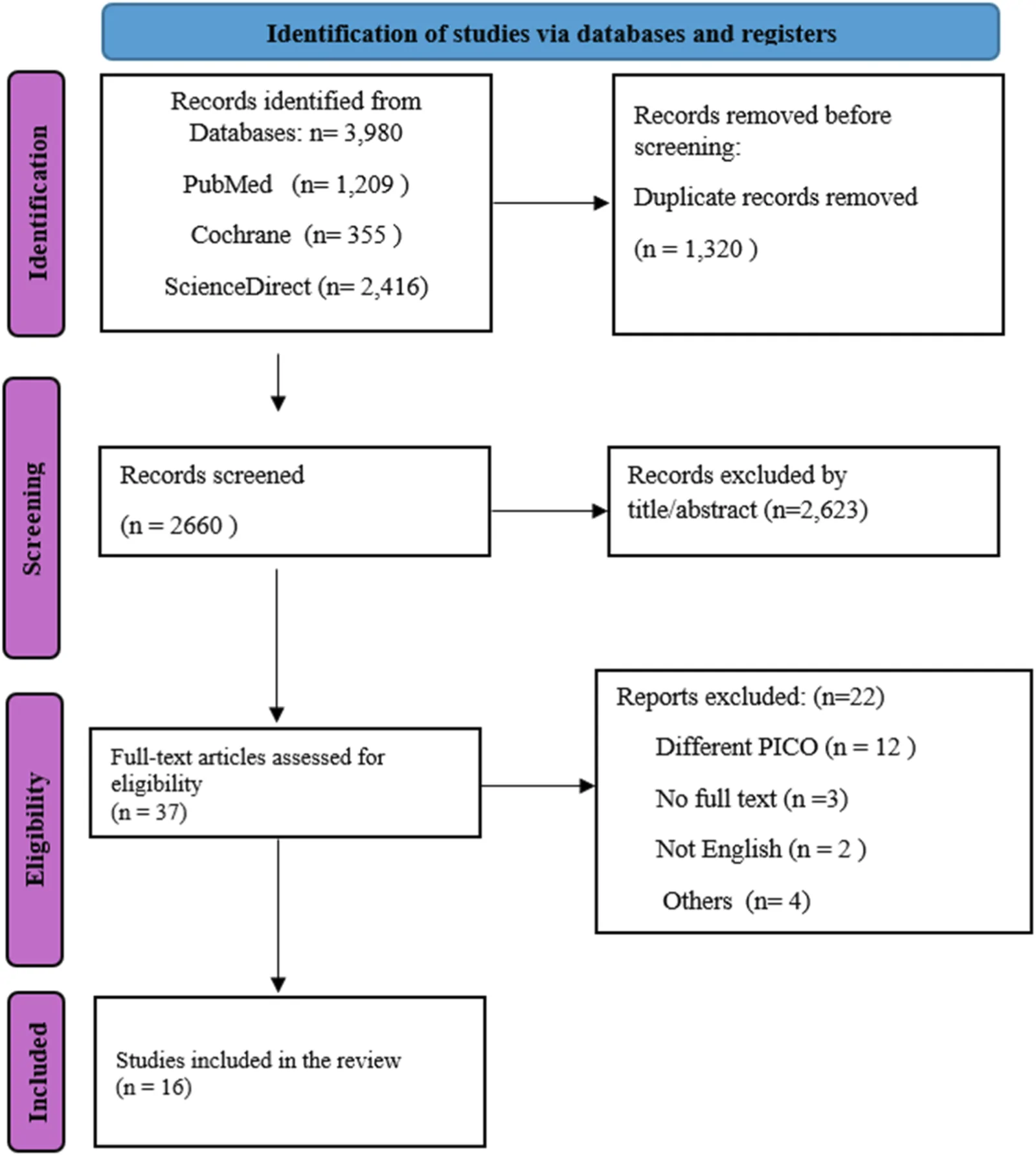
PRISMA flowchart of the screening process.

A total of 3,980 records were identified through database searches, of which 2,660 remained after deduplication (Figure 1). Following title and abstract screening, 2,621 articles were excluded, leaving 37 full-text studies assessed for eligibility. Ultimately, 22 studies were excluded due to failure to meet inclusion criteria, resulting in 16 included studies in this review (Fujieda et al., 2020; ; Jonstam et al., 2019; Maspero et al., 2020; Bachert et al., 2016; Bachert et al., 2017; Han et al., 2021; Boguniewicz et al., 2021; Canonica et al., 2022a; Tversky et al., 2021; Canonica et al., 2022b; Takabayashi et al., 2021; Bachert et al., 2022a; Gevaert et al., 2020; Hayashi et al., 2020; De Corso et al., 2025) (Figure 1).

### Study characteristics

A total of 16 studies involving 4,861 patients. All of the studies were randomized controlled trials (RCTs). Treatment durations ranged from 16 weeks to 52 weeks, with the majority spanning 24–52 weeks. Geographically, 5 studies were conducted in the USA, 3 in Japan, 4 in Belgium, and the remaining studies were multinational (e.g., North America/Europe) or conducted in Argentina. Common comorbidities included asthma, allergy/atopy, and NSAID-exacerbated respiratory disease. Baseline symptoms such as nasal congestion (mean scores ranging from 1.61 to 9.06) and loss of smell (UPSIT scores ranging from 10.7 to 15.2) were frequently documented. Prior endoscopic surgery was reported in 8 studies, with proportions ranging from 58% to 100%. The Sino-Nasal Outcome Test-22 (SNOT-22) scores varied between 31.3 and 69.3, indicating moderate to severe symptom burden at baseline.

Baseline demographic and clinical characteristics of patients are detailed in (Table 1).

**TABLE 1:** Summary of the included studies.

Study, year	Country	Treatment	Loading dose	Maintenance dose	Frequency	Treatment duration	Sample size
Fujieda 2021	Japan	Dupilumab	600 mg	300 mg	q2w	52 weeks	16
		Dupilumab	600 mg	300 mg	q2w–q4w	52 weeks	17
		Placebo	—	—	—	52 weeks	16
Bachert 2019 (SINUS-24) and (SINUS-52)	Belgium	Dupilumab	600 mg	300 mg	q2w	24 weeks	143
		Placebo	—	—	—	24 weeks	133
		Dupilumab	600 mg	300 mg	q2w	24 weeks	150
		Dupilumab	600 mg	300 mg	q2w–q4w	24 weeks	145
		Placebo	—	—	—	24 weeks	153
Jonstam 2019	USA	Dupilumab + MFNS	600 mg	300 mg	q2w–q4w	16 weeks	30
		Placebo + MFNS	—	—	—	16 weeks	30
Maspero 2020	Argentina	Dupilumab	400 mg	200 mg	q2w	52 weeks	126
		Placebo	—	—	—	52 weeks	63
		Dupilumab	600 mg	300 mg	q2w	52 weeks	123
		Placebo	—	—	—	52 weeks	70
Bachert 2016	US/ Europe	Dupilumab	600 mg	300 mg	Weekly	16 weeks	30
		Placebo	—	—	—	16 weeks	30
Bachert 2017	Belgium	Mepolizumab	—	750 mg	q4w	25 weeks	54
		Placebo	—	—	—	25 weeks	51
Han 2021 (SYNAPSE)	USA	Mepolizumab	—	100 mg	q4w	52 weeks	206
		Placebo	—	—	—	52 weeks	201
Boguniewicz 2021	USA	Dupilumab	600 mg	300 mg	q2w	16 weeks	348
		Dupilumab	600 mg	300 mg	Weekly	16 weeks	434
		Placebo	—	—	—	16 weeks	389
Harrison 2021 (OSTRO)	Multinational	Benralizumab	—	30 mg	q8w (first 3 doses q4w)	24 weeks	427
		Placebo	—	—	—	24 weeks	229
Tversky 2021	USA	Benralizumab	—	30 mg	q4w	20 weeks	12
		Placebo	—	—	—	20 weeks	12
Canonica 2022	Italy	Dupilumab	600 mg	300 mg	q2w	24 weeks	78
		Placebo	—	—	—	24 weeks	38
Takabayashi 2021	Japan	Benralizumab	—	30 mg	Single dose	24 weeks	22
		Benralizumab	—	30 mg	q8w	24 weeks	23
		Placebo	—	—	—	24 weeks	11
Bachert 2022	Belgium	Benralizumab	—	30 mg	q8w (first 3 doses q4w)	40 weeks	207
		Placebo	—	—	—	40 weeks	203
Gevaert 2020 (POLYP-1) and (POLYP-2)	North America & europe	Omalizumab	—	75–600 mg	q2w or q4w	24 weeks	138
		Placebo	—	—	—	24 weeks	—
		Omalizumab	—	75–600 mg	q2w or q4w	24 weeks	127
		Placebo	—	—	—	24 weeks	—
Hayashi 2020	Japan	Omalizumab	—	300 mg	q2w–q4w	18 weeks	16
De Corso 2025	Italy	Dupilumab	600 mg	300 mg	q2w	24 weeks	24
		Omalizumab	—	75–600 mg	q2w–q4w	24 weeks	24

SC: subcutaneous, q2w: every 2 weeks, q4w: every 4 weeks, qw: every week, mg: milligram.

### Risk of bias assessment

**FIGURE 2:**
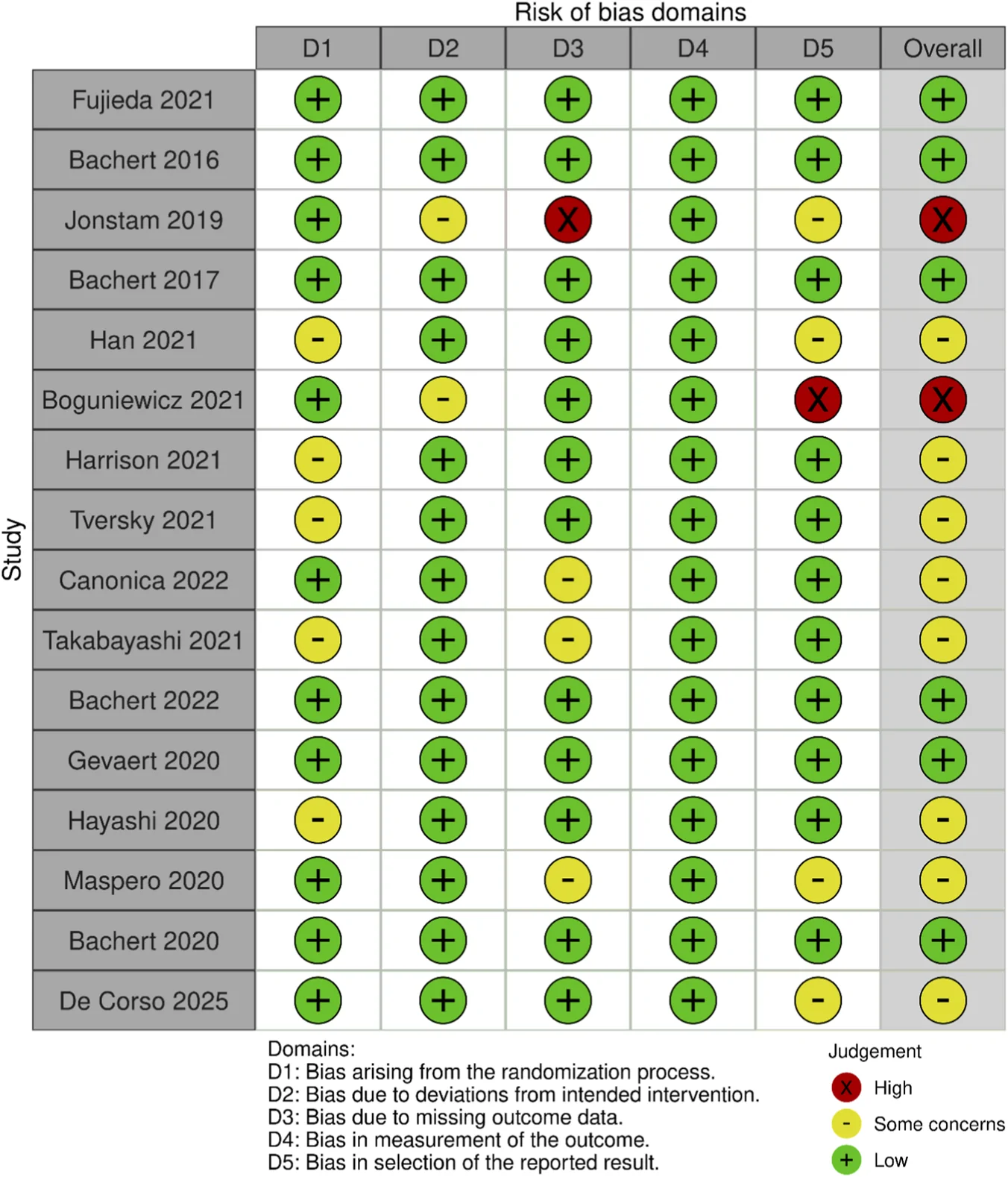
Detailed Risk of Bias (RoB 2) assessment for each of the included randomized controlled trials.

**FIGURE 3:**
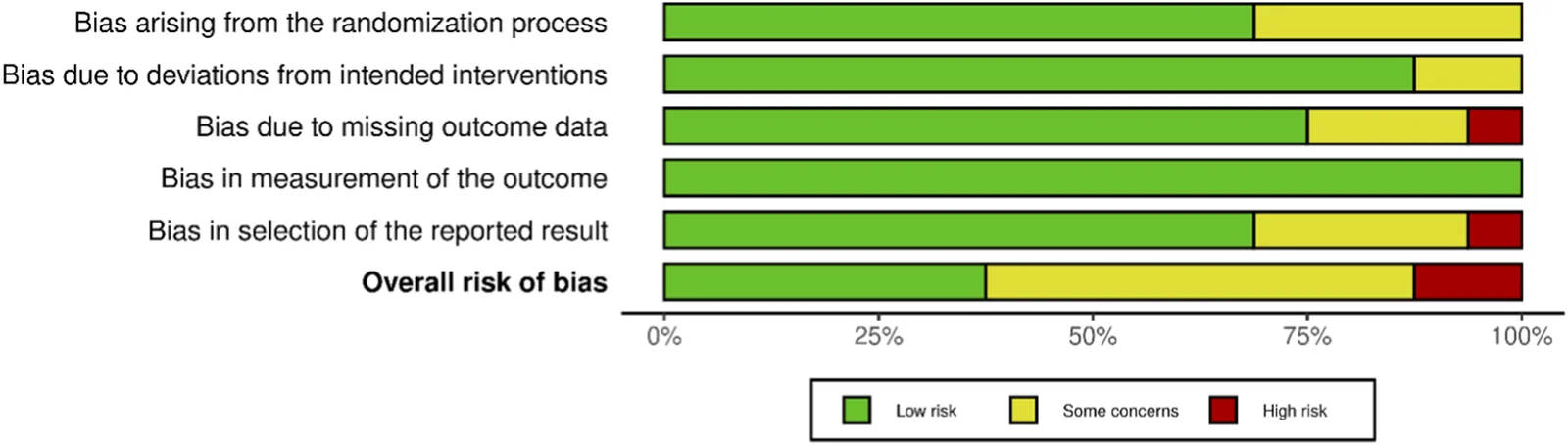
Summary of overall ROB of the included studies.

The risk of bias (ROB) analysis using the ROB-2 tool across 16 studies revealed 5 studies with low risk, 2 with high risk, and 8 with some concerns. High-risk studies were primarily flagged for missing outcome data and selective reporting. Most trials demonstrated robust methodology, including proper randomization and outcome measurement, though deviations in blinding or protocol adherence were noted in some. Overall, while the majority of findings are reliable, results from high-risk studies should be interpreted cautiously due to potential biases affecting validity (Figures 2, 3). Assessments were independently performed.

### Nasal polyp score (NPS)

**FIGURE 4:**
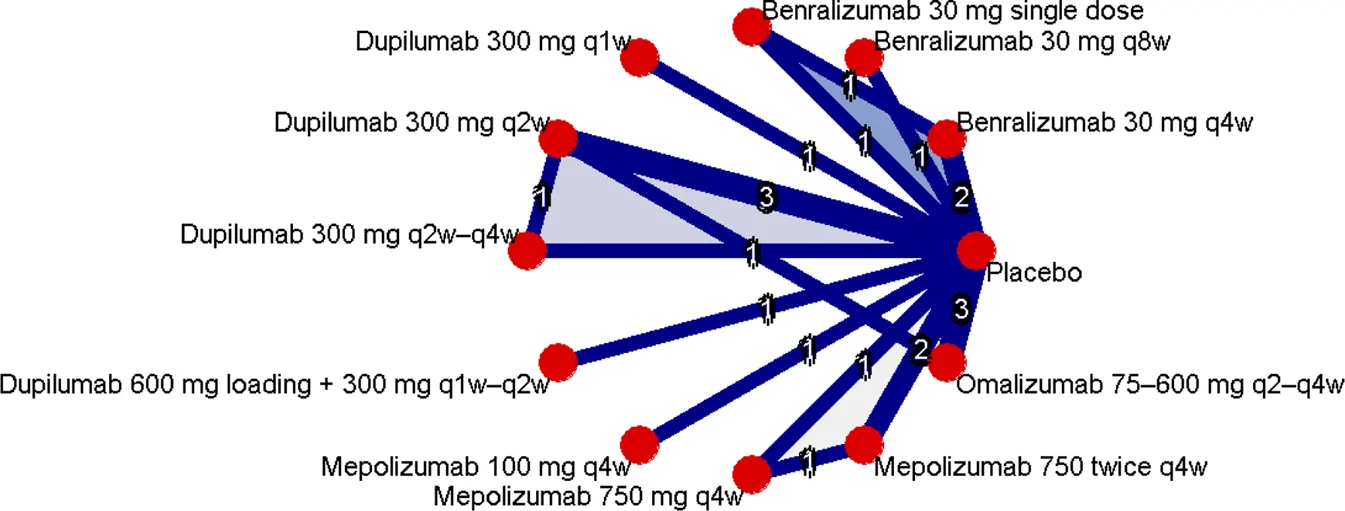
Network geometry of treatment comparisons for Nasal Polyp Score (NPS).

The network meta-analysis for NPS included 21 direct comparisons derived from randomized controlled trials evaluating dupilumab, omalizumab, mepolizumab, benralizumab, and placebo. Across the network, biologic therapies generally demonstrated greater reductions in NPS than placebo (Figure 4).

### Treatment ranking

**FIGURE 5:**
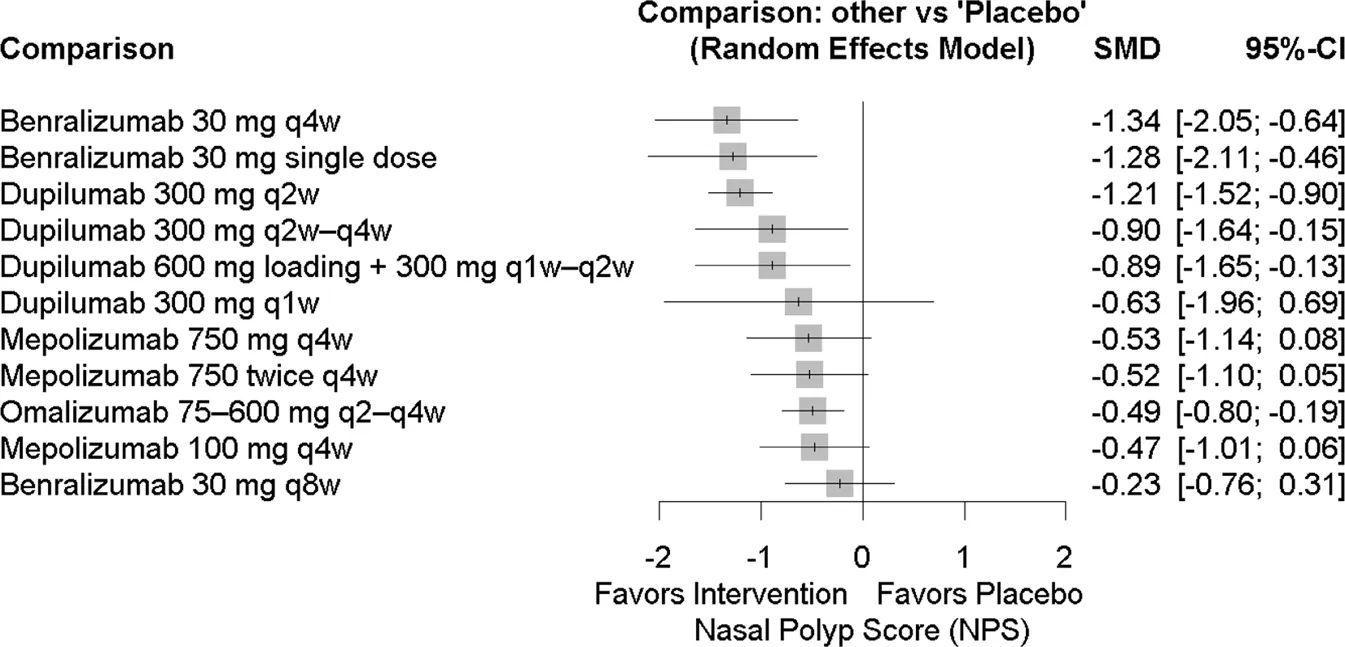
Forest plot of network meta-analysis estimates for Nasal Polyp Score (NPS).

According to the random-effects P-scores, the highest-ranked interventions for reducing NPS were benralizumab 30 mg every 4 weeks (P-score = 0.864), dupilumab 300 mg every 2 weeks (P-score = 0.829), and benralizumab 30 mg single dose (P-score = 0.822). Intermediate rankings were observed for dupilumab 300 mg every 2–4 weeks (P-score = 0.628), dupilumab 600 mg loading followed by 300 mg every 1–2 weeks (P-score = 0.625), and dupilumab 300 mg weekly (P-score = 0.467) (Figure 5).

### Heterogeneity and inconsistency

The network demonstrated moderate heterogeneity (τ = 0.254, τ^2^ = 0.065, I^2^ = 68.9%). Global inconsistency assessment using the design-by-treatment interaction model showed significant inconsistency across the network (Q = 22.57, df = 7, p = 0.002), with evidence of both within-design heterogeneity (Q = 12.39, df = 3, p = 0.006) and between-design inconsistency (Q = 10.19, df = 4, p = 0.037).

**FIGURE 6:**
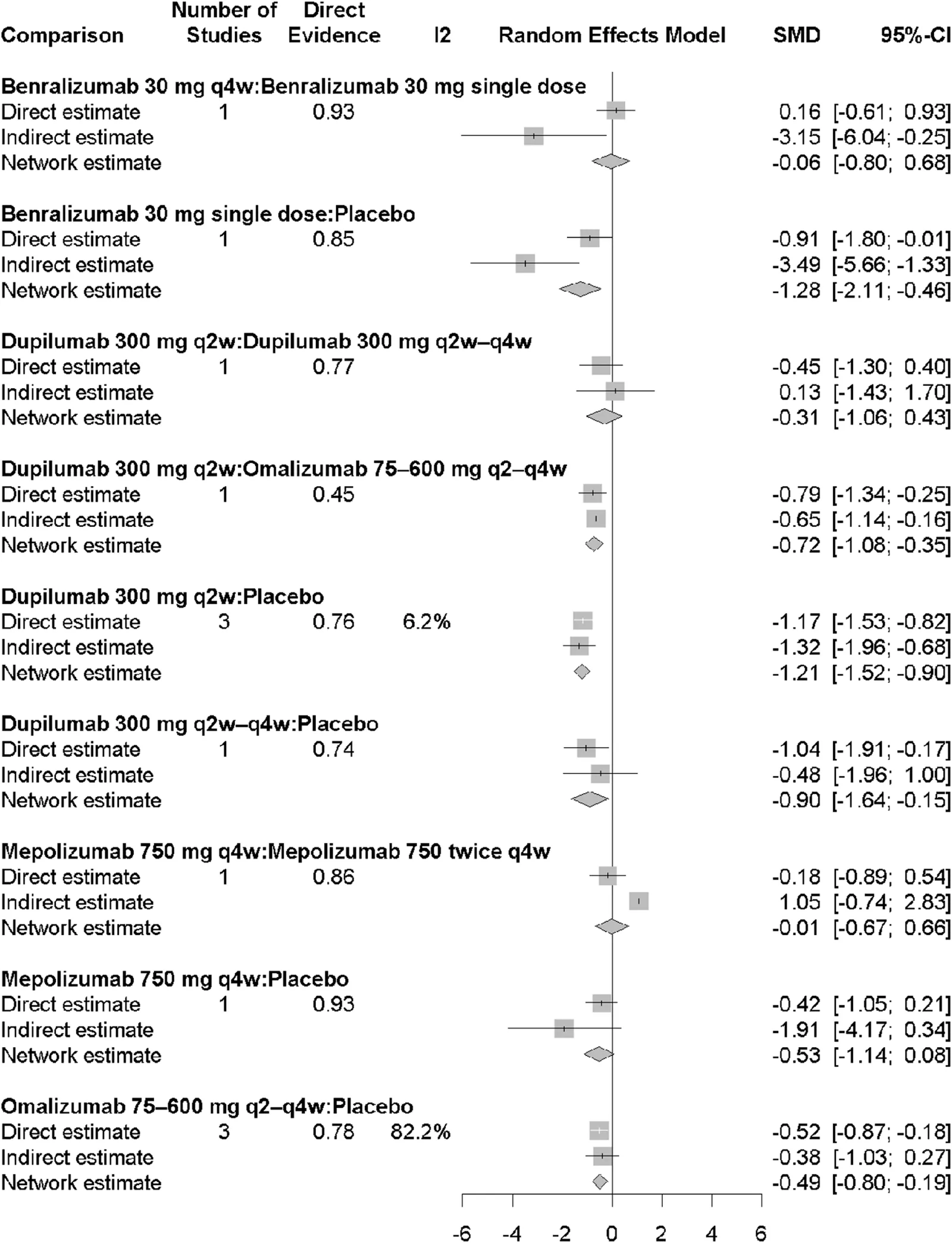
Node-splitting analysis assessing local inconsistency in the network meta-analysis of Nasal Polyp Score (NPS).

Node-splitting analyses identified significant local inconsistency involving comparisons between benralizumab 30 mg every 4 weeks and benralizumab single-dose regimens (p = 0.031), as well as between benralizumab single dose and placebo (p = 0.031). No significant inconsistency between direct and indirect evidence was observed for comparisons involving dupilumab, omalizumab, or mepolizumab (all p > 0.05) (Figure 6) (Supplementary Figure S1).

### Small-study effects

**FIGURE 7:**
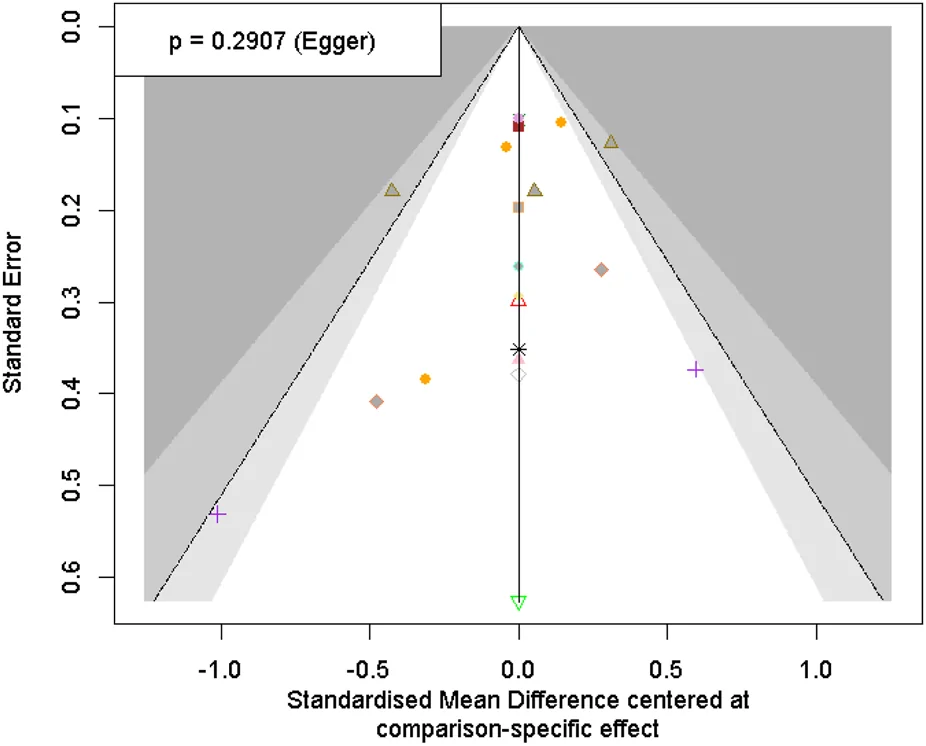
Comparison-adjusted funnel plot for assessment of small-study effects in the network meta-analysis of Nasal Polyp Score (NPS).

Egger’s regression test showed no evidence of publication bias or small-study effects (t = −1.09, p = 0.291) (Figure 7).

### Sino-Nasal Outcome Test-22 (SNOT-22)

**FIGURE 8:**
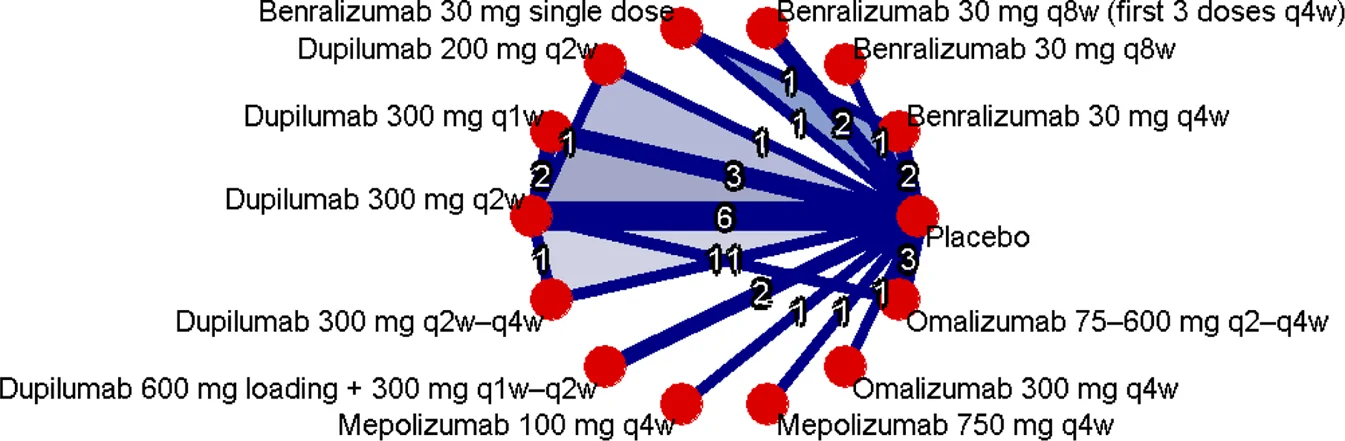
Network geometry of treatment comparisons for Sino-Nasal Outcome Test-22 (SNOT-22).

The SNOT-22 network comprised 31 direct treatment comparisons involving multiple dosing regimens of dupilumab, omalizumab, mepolizumab, benralizumab, and placebo (Figure 8).

### Treatment ranking

**FIGURE 9:**
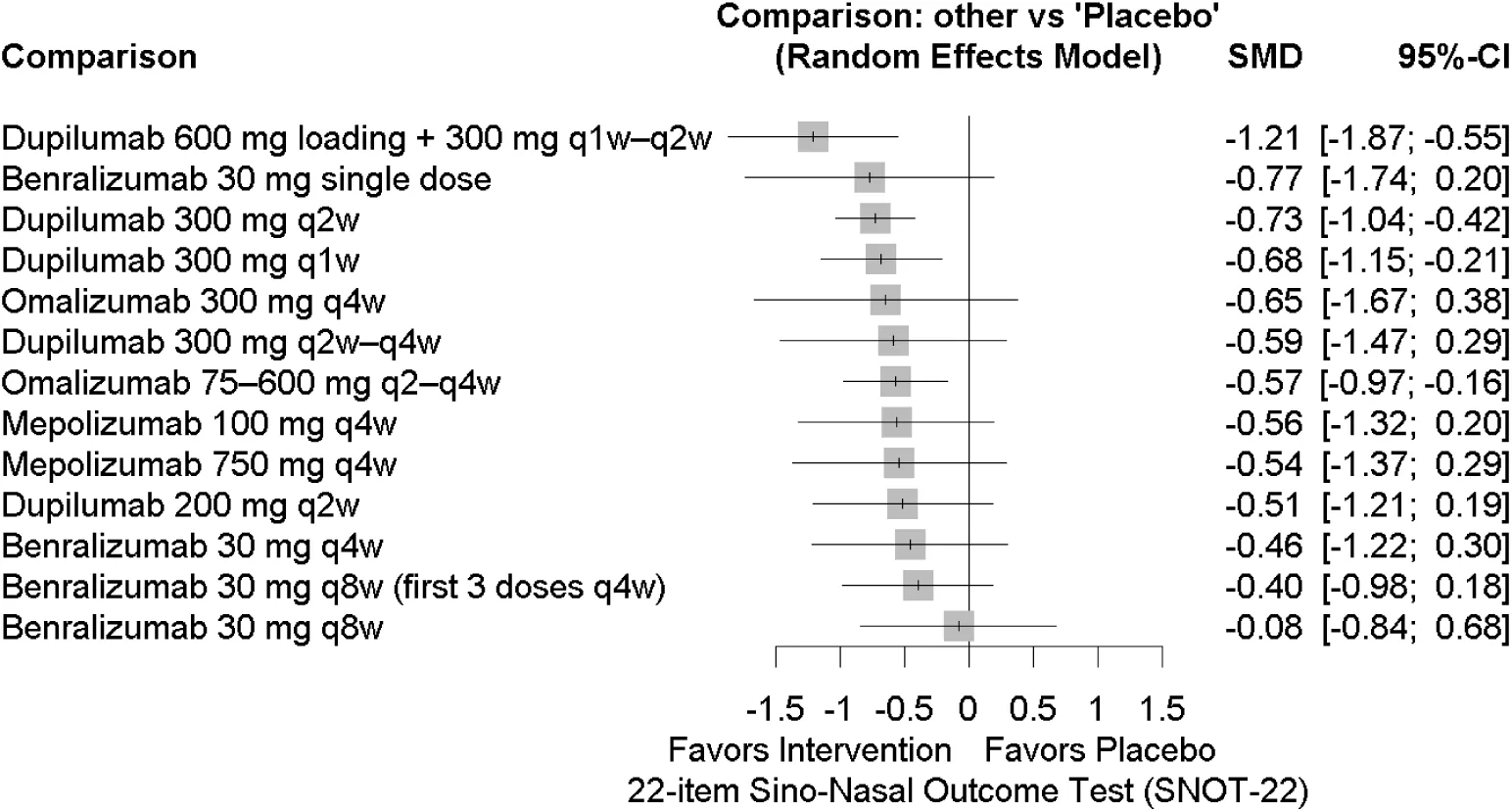
Forest plot of network meta-analysis estimates for Sino-Nasal Outcome Test-22 (SNOT-22).

Under the random-effects model, dupilumab 600 mg loading followed by 300 mg every 1–2 weeks ranked as the most effective intervention for improving SNOT-22 scores (P-score = 0.907). Other highly ranked treatments included dupilumab 300 mg every 2 weeks (P-score = 0.666), benralizumab 30 mg single dose (P-score = 0.644), and dupilumab 300 mg weekly (P-score = 0.611). Omalizumab 300 mg every 4 weeks (P-score = 0.561), dupilumab 300 mg every 2–4 weeks (P-score = 0.526), mepolizumab 100 mg every 4 weeks (P-score = 0.512), and omalizumab 75–600 mg every 2–4 weeks (P-score = 0.511) demonstrated moderate efficacy (Figure 9).

### Heterogeneity, inconsistency, & publication bias

High heterogeneity was observed across the network (τ = 0.374, τ^2^ = 0.140, I^2^ = 86.5%). Global inconsistency testing showed inconsistency between direct and indirect evidence (Q = 96.53, df = 13, p < 0.001). Significant within-design heterogeneity (Q = 14.11, df = 7, p = 0.049) and marked between-design inconsistency (Q = 82.42, df = 6, p < 0.001) were identified.

**FIGURE 10:**
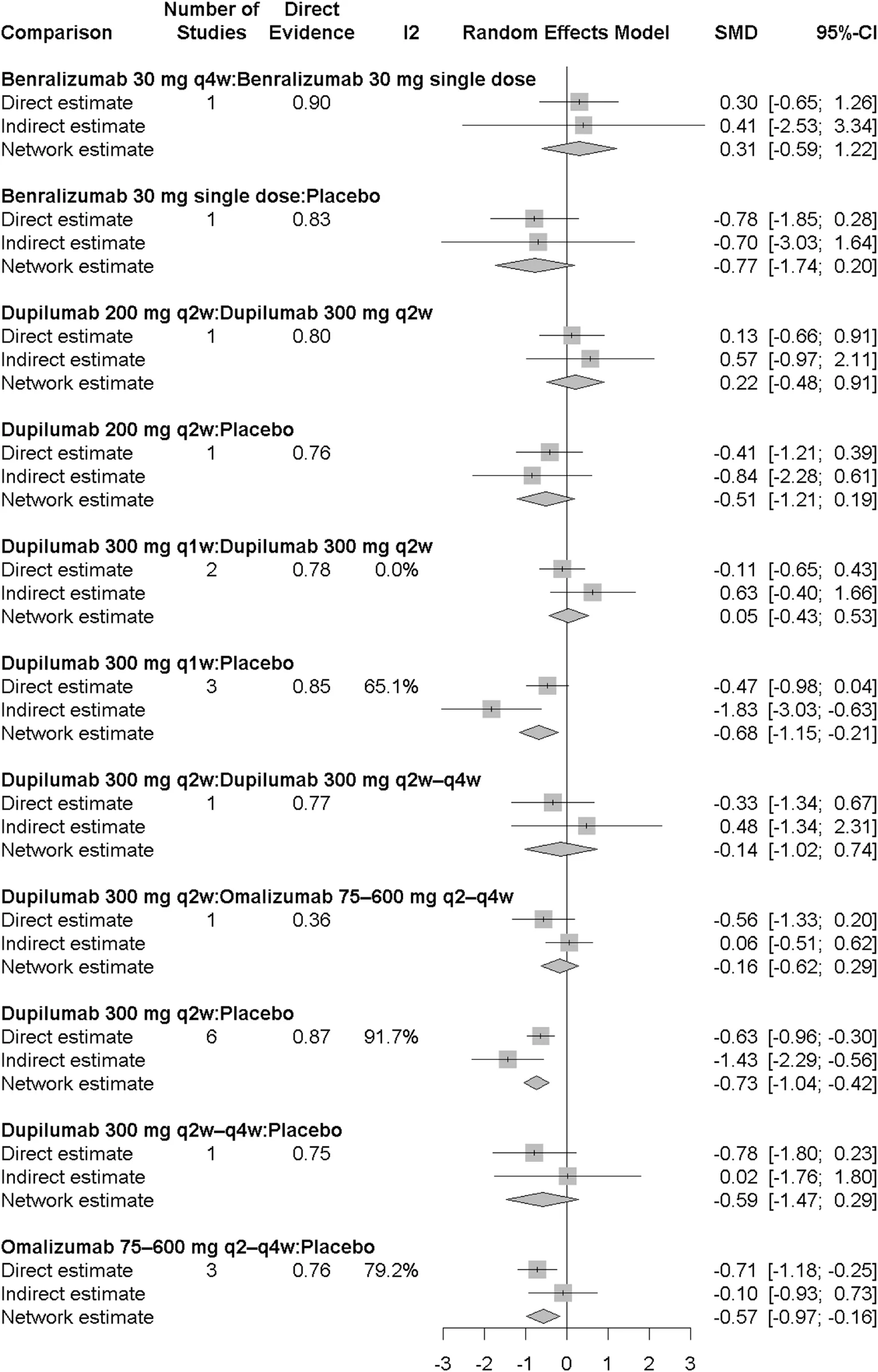
Node-splitting analysis assessing local inconsistency in the network meta-analysis of Sino-Nasal Outcome Test-22 (SNOT-22).

Design-specific analyses suggested that studies comparing omalizumab with placebo contributed most strongly to network heterogeneity (Q = 9.61, p = 0.008). Node-splitting analyses further demonstrated significant local inconsistency for several comparisons, including dupilumab 300 mg weekly versus dupilumab 300 mg every 2 weeks (p < 0.001), dupilumab 300 mg weekly versus placebo (p < 0.001), dupilumab 300 mg every 2 weeks versus omalizumab 75–600 mg every 2–4 weeks (p < 0.001), and dupilumab 300 mg every 2 weeks versus placebo (p < 0.001) (Figure 10) (Supplementary Figure S2).

**FIGURE 11:**
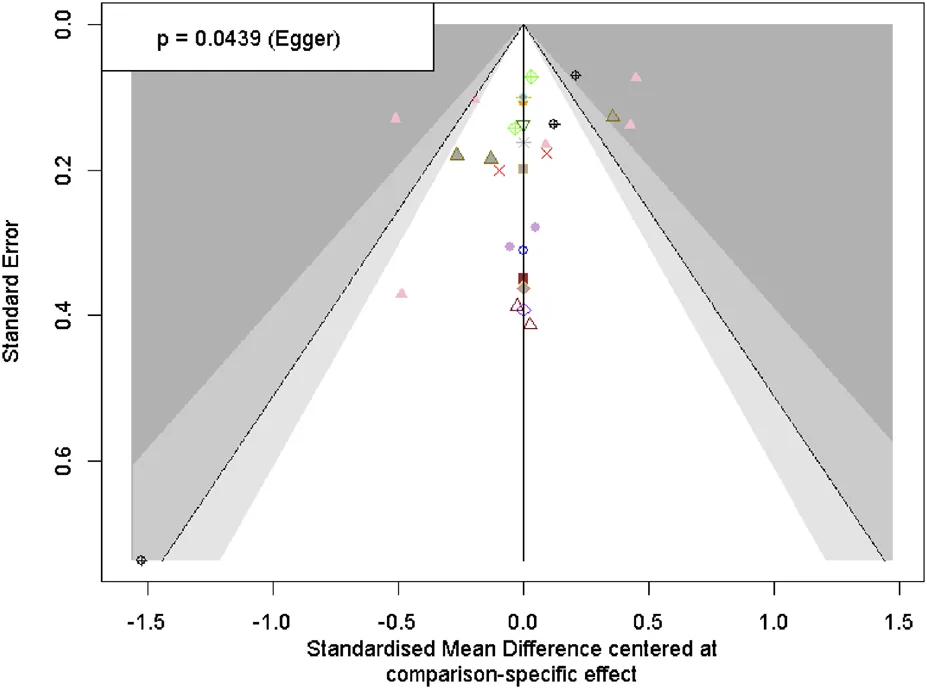
Comparison-adjusted funnel plot for assessment of small-study effects in the network meta-analysis of Sino-Nasal Outcome Test-22 (SNOT-22).

Egger’s regression test showed evidence of publication bias or small-study effects (t = −2.11, p = 0.0439) (Figure 11).

### University of Pennsylvania Smell Identification Test (UPSIT)

**FIGURE 12:**
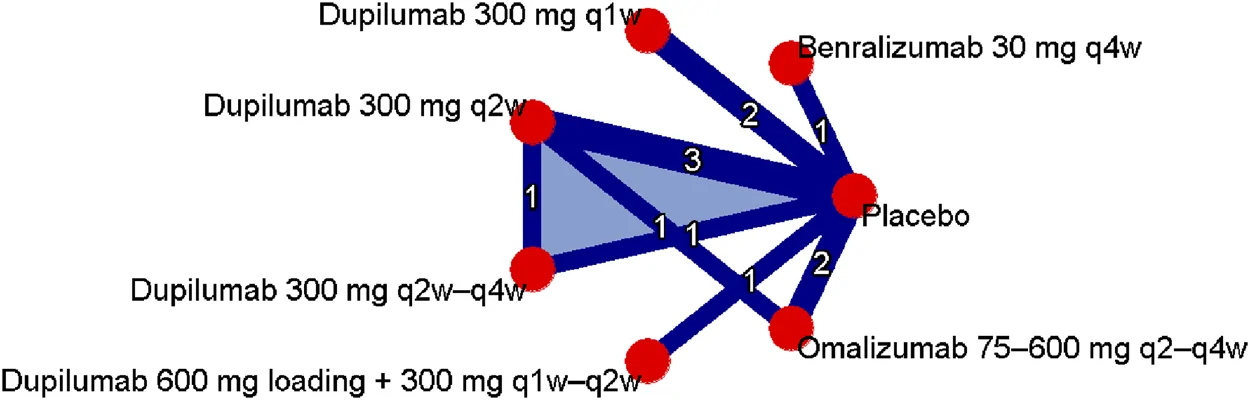
Network geometry of treatment comparisons for University of Pennsylvania Smell Identification Test (UPSIT).

The UPSIT network included 12 direct comparisons evaluating dupilumab, omalizumab, benralizumab, and placebo (Figure 12).

### Treatment effects and ranking

Network estimates showed significant improvements in olfactory function for multiple biologic therapies compared with placebo. The largest effect size was observed with dupilumab 600 mg loading followed by 300 mg every 1–2 weeks (SMD = 1.91, 95% CI 1.24–2.59; p < 0.001), followed by dupilumab 300 mg every 2 weeks, dupilumab 300 mg every 2–4 weeks, and omalizumab 75–600 mg every 2–4 weeks.

**FIGURE 13:**
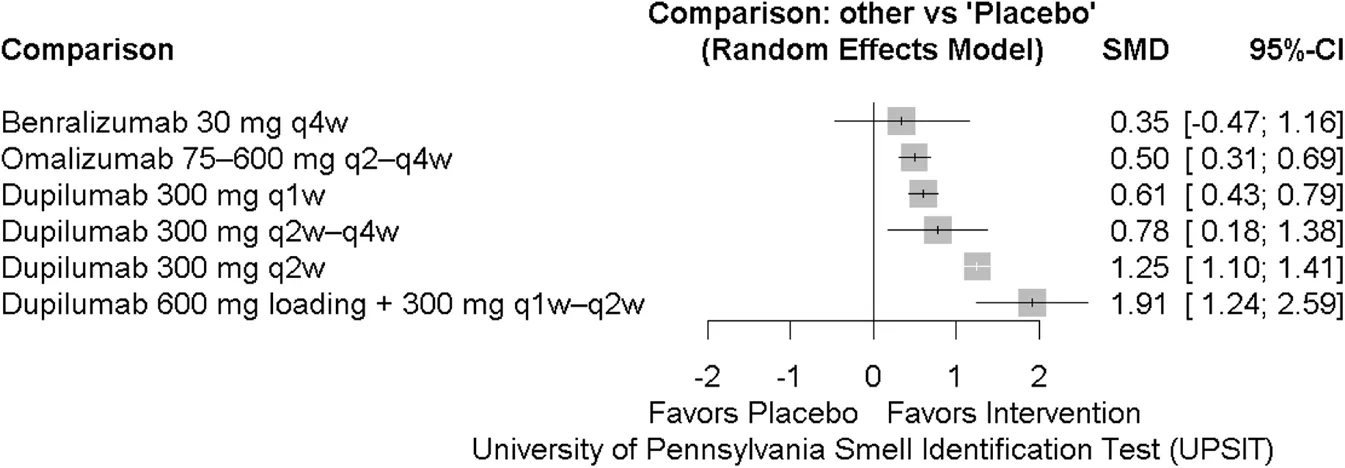
Forest plot of network meta-analysis estimates for University of Pennsylvania Smell Identification Test (UPSIT).

Because UPSIT scores were analyzed in reverse order, lower P-scores indicate greater efficacy. Accordingly, dupilumab 600 mg loading followed by 300 mg every 1–2 weeks ranked as the most effective treatment (P-score = 0.006), followed by dupilumab 300 mg every 2 weeks (P-score = 0.175), dupilumab 300 mg every 2–4 weeks (P-score = 0.436), dupilumab 300 mg weekly (P-score = 0.530), omalizumab 75–600 mg every 2–4 weeks (P-score = 0.662), and benralizumab 30 mg every 4 weeks (P-score = 0.726) (Figure 13).

### Heterogeneity, Inconsistency, & Publication Bias

The UPSIT network demonstrated minimal heterogeneity (τ = 0.048, τ^2^ = 0.002, I^2^ = 7.5%). Global inconsistency testing showed no evidence of inconsistency (Q = 5.40, df = 5, p = 0.369), with neither within-design heterogeneity (Q = 3.40, df = 3, p = 0.334) nor between-design inconsistency (Q = 2.01, df = 2, p = 0.367) reaching statistical significance.

**FIGURE 14:**
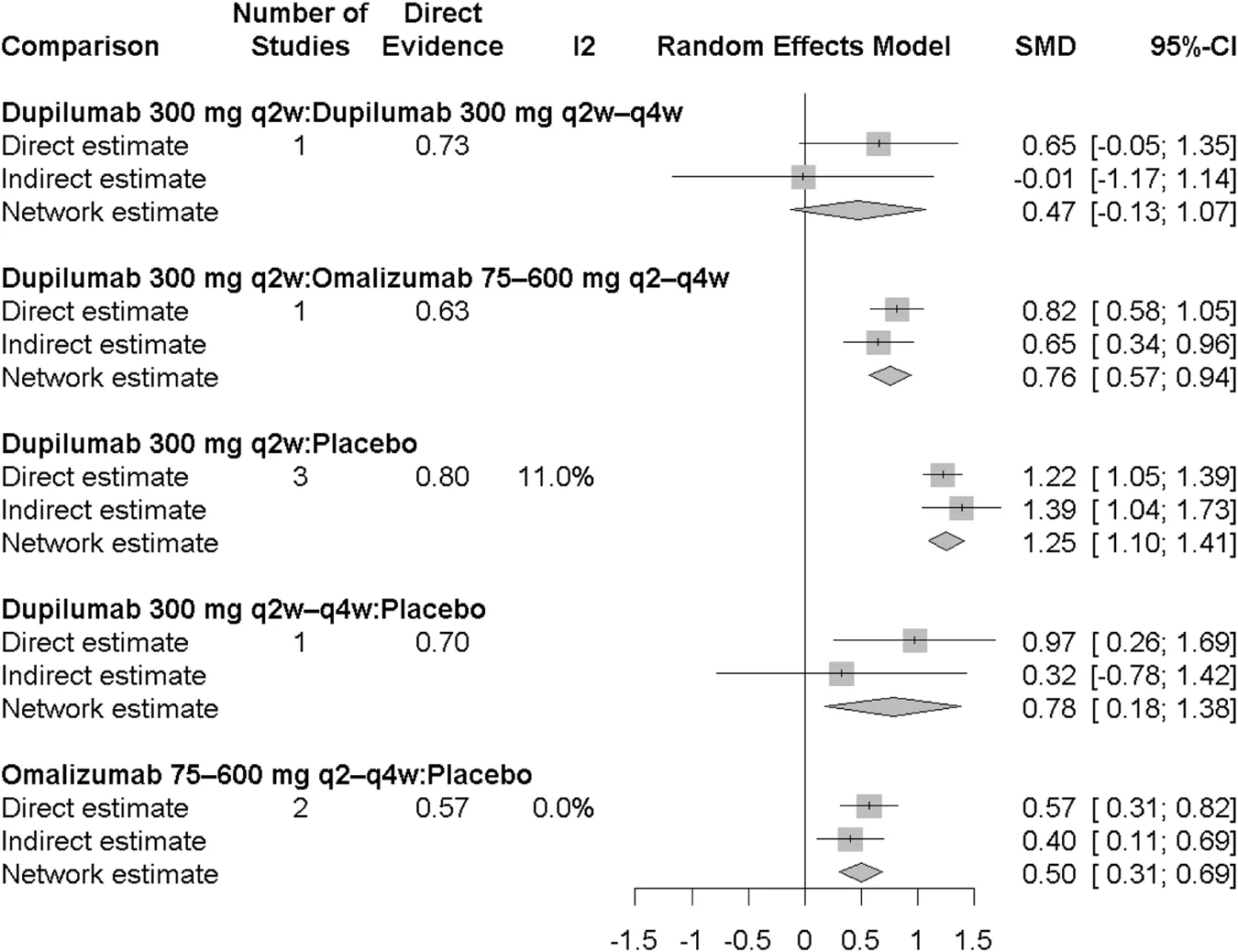
Node-splitting analysis assessing local inconsistency in the network meta-analysis of University of Pennsylvania Smell Identification Test (UPSIT).

Node-splitting analyses confirmed consistency between direct and indirect evidence for all evaluated treatment comparisons, including dupilumab versus placebo, dupilumab versus omalizumab, and dupilumab dosing comparisons (all p > 0.05) (Figure 14) (Supplementary Figure S3).

**FIGURE 15:**
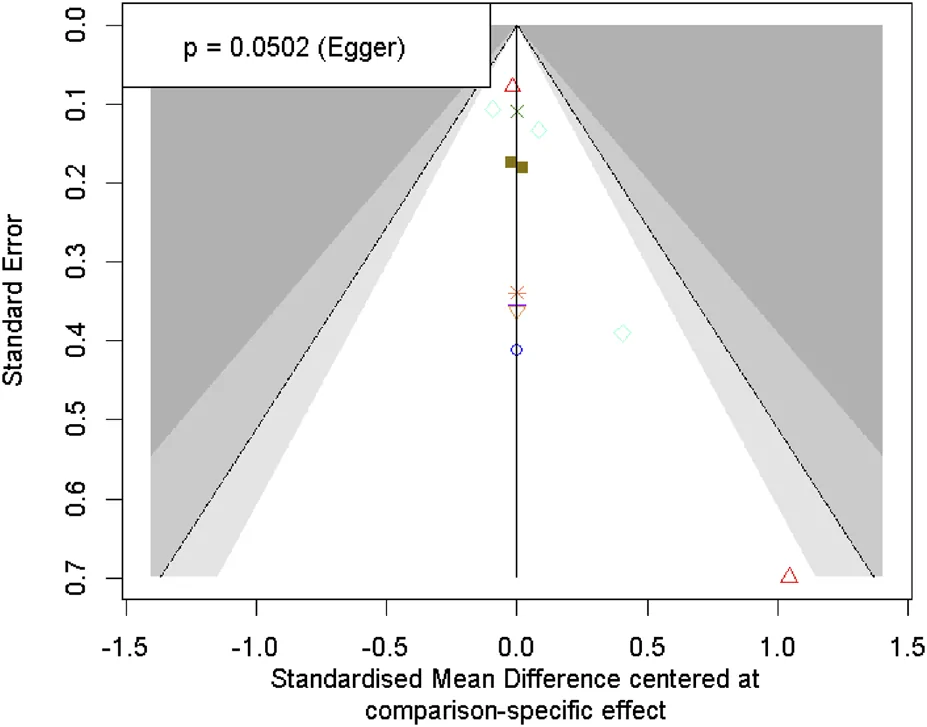
Comparison-adjusted funnel plot for assessment of small-study effects in the network meta-analysis of University of Pennsylvania Smell Identification Test (UPSIT).

Egger’s regression test showed some evidence of publication bias or small-study effects (t = 2.23, p = 0.0502) (Figure 15).

## Discussion

Biologic therapies targeting type 2 inflammation, including dupilumab, omalizumab, benralizumab, and mepolizumab, have shown significant benefits in patients with chronic rhinosinusitis with nasal polyps (CRSwNP) compared with placebo overall, but their relative efficacy varies by outcome and patient phenotype. Prior systematic reviews of biologics in CRSwNP evaluated efficacy by drug but did not compare differences in dose or frequency. On the other hand, our network meta-analysis included multiple available doses and frequencies of regimens for each biologic, allowing comparison of different regimens and their relative efficacy, a component not explored in previous meta-analyses. Although biologic therapy ranked differently across outcomes, the confidence intervals for numerous treatment estimates overlapped significantly. This shows that, while some regimens appear to be better based on ranking measures like P-scores, the underlying clinical differences between different biologic therapies may be minor. Therefore, treatment selection should also consider individual patient characteristics, comorbidities, safety profiles, and treatment preferences in addition to comparative efficacy.

### SNOT-22 (quality of life)

Dupilumab provided the greatest benefit for patient-reported sinonasal quality of life in our network meta-analysis. Dupilumab with a 600 mg loading dose followed by 300 mg every 1–2 weeks resulted in an SMD = −1.21 (95% CI –1.87 to −0.55) versus placebo. According to the random-effects P-score rankings, this regimen demonstrated the highest efficacy (P-score = 0.907), followed by dupilumab 300 mg every 2 weeks (P-score = 0.666), and benralizumab 30 mg single dose (P-score = 0.644). These results are in line with other research showing that dupilumab therapy significantly improves quality of life. Recent network meta-analyses and clinical guidelines similarly identify dupilumab as a leading biologic option for improving SNOT-22 outcomes in CRSwNP (Rank et al., 2023; Wang et al., 2025). Improvements in key patient-reported symptoms, including nasal obstruction and loss of smell, have also been documented in clinical studies evaluating dupilumab (Al-Ahmad et al., 2025). Nevertheless, the SNOT-22 network exhibited substantial heterogeneity and significant inconsistency between direct and indirect evidence. Therefore, although dupilumab ranked highest, the magnitude of superiority over competing biologics should be interpreted with caution.

### Nasal polyp score (NPS)

The biggest reduction in polyp load was shown by benralizumab 30 mg every 4 weeks (SMD –1.34; 95% CI –2.05 to −0.64). According to the random-effects P-score rankings, benralizumab 30 mg every 4 weeks was the most effective agent (P-score = 0.864), followed by dupilumab 300 mg every 2 weeks (P-score = 0.829), and benralizumab 30 mg single dose (P-score = 0.822). Despite modest effect sizes, mepolizumab and omalizumab both significantly reduced NPS compared with placebo. These results are consistent with findings from major clinical trials and recent meta-analyses demonstrating significant reductions in polyp size with biologic therapy, particularly with benralizumab and dupilumab (Bachert et al., 2019; Wang et al., 2025; Bachert et al., 2022b). These results reveal that both IL-4/13 blockade (dupilumab) and IL-5 pathway inhibition (benralizumab, mepolizumab) are effective at reducing polyp burden, with benralizumab demonstrating very strong potency. However, the NPS network showed moderate heterogeneity and significant inconsistency, suggesting that treatment rankings should be interpreted cautiously.

### Olfactory function (UPSIT)

Olfactory outcomes were most improved with dupilumab. Network estimates showed significant improvements in olfactory function for multiple biologic therapies compared with placebo. Because the UPSIT network was analyzed using reverse ranking, lower P-scores indicate greater efficacy; therefore, dupilumab 600 mg loading followed by 300 mg every 1–2 weeks (P-score = 0.006) ranked as the most effective treatment, followed by dupilumab 300 mg every 2 weeks (P-score = 0.175), and dupilumab 300 mg every 2–4 weeks (P-score = 0.436). Omalizumab also significantly improved UPSIT scores, while benralizumab did not achieve statistical significance (SMD 0.35; 95% CI −0.47 to 1.16).

This lack of statistical significance with benralizumab is consistent with prior network meta-analyses, which suggested that its effect on symptom-based outcomes was more modest than that of other biologics (Wang et al., 2025). There could be several reasons for these results. By targeting the IL-5 receptor, benralizumab reduces the number of circulating eosinophils; however, it has little effect on residual tissue eosinophils or on other inflammatory pathways, such as IL-4/IL-13 and IgE-mediated reactions. Compared with dupilumab, which directly suppresses IL-4 and IL-13, this restricted range of action may contribute to a less pronounced effect on olfactory recovery compared with therapies targeting broader type-2 inflammatory pathways. In contrast to the SNOT-22 and NPS networks, the UPSIT network demonstrated low heterogeneity and no significant inconsistency, thereby increasing confidence in the observed superiority of dupilumab for olfactory recovery (Wang et al., 2025; Bachert et al., 2022b).

Our findings suggest that dosing regimens may influence clinical outcomes across biologic therapies in CRSwNP. Dupilumab 600 mg loading followed by 300 mg every 1–2 weeks showed the greatest improvement in patient-reported quality of life and olfactory function, suggesting that higher-frequency dosing may be associated with greater symptom improvement and recovery of olfactory function. Benralizumab, on the other hand, reduced the nasal polyp score the most when given at a dose of 30 mg every 4 weeks. This suggests that different biologics may produce the best structural results when given in different ways. It is interesting to note that dupilumab given every 2 weeks and then every 4 weeks maintained a therapeutic effect, indicating that longer dose intervals may preserve efficacy once disease management is achieved. This observation is consistent with emerging real-world evidence showing that patients with CRSwNP who transitioned from dupilumab every 2 weeks to every 4 weeks maintained symptom control and nasal polyp outcomes after achieving stable disease control. These findings are also consistent with earlier research showing that dupilumab significantly improves olfactory function in patients with CRSwNP. (Lane et al., 2025). Biologic treatments, especially dupilumab, are highlighted in current clinical guidelines as crucial choices for scent restoration in cases of severe disease (Whitcroft et al., 2023).

Recently, the EVEREST trial provided a direct comparison of dupilumab and omalizumab in patients with CRSwNP and further supported the strong clinical efficacy of dupilumab across key disease outcomes (De Corso et al., 2025). This evidence is broadly consistent with the treatment rankings observed in our analysis. In addition, new biologic treatments that target different inflammatory pathways are also being researched. The phase 3 WAYPOINT trial demonstrated that tezepelumab significantly improved nasal polyp score, nasal congestion, and quality-of-life outcomes in severe CRSwNP. As an upstream inhibitor of thymic stromal lymphopoietin (TSLP), it targets a broader inflammatory pathway than currently approved biologics. Although not included in this network meta-analysis because it fell outside the predefined interventions, its promising efficacy warrants evaluation in future comparative analyses against established biologics (Lipworth et al., 2025). These findings further underscore the rapidly evolving therapeutic landscape of biologic treatment for CRSwNP.

Overall, these results suggest that while all biologics improve disease outcomes, their relative benefits vary by clinical endpoint, with dupilumab showing stronger effects on symptom-based outcomes and benralizumab demonstrating greater reductions in objective polyp burden.

### Sensitivity analyses and publication bias

Two trials were found to have a high risk of bias, primarily due to incomplete or biased reporting. SNOT-22, NPS, and UPSIT effect estimates were not significantly changed by excluding these investigations, and the relative ranking of biologics did not change. Heterogeneity varied across outcomes, being high for SNOT-22, moderate for NPS, and low for UPSIT.

Global inconsistency testing identified significant inconsistency in both the SNOT-22 and NPS networks, whereas the UPSIT network remained consistent. Funnel plot assessment and Egger’s regression suggested possible small-study effects for SNOT-22, no evidence of publication bias for NPS, and borderline evidence for UPSIT. Despite these findings, sensitivity analyses demonstrated stable treatment rankings after exclusion of high-risk-of-bias studies, supporting the overall robustness of the results.

### Long-term considerations

Most randomized trials included in this analysis evaluated outcomes over 24–52 weeks; however, emerging evidence suggests that biologic therapies may maintain efficacy beyond 1 year. Recent reviews and meta-analyses have found that dupilumab consistently improves nasal polyp score, quality of life, and olfactory function over extended follow-up periods, with no significant differences in major adverse events between biologics. (Chen et al., 2025; Cai et al., 2025). These findings support the use of biologics as a maintenance therapy for people with severe CRSwNP who do not respond to standard treatments. Nonetheless, questions remain about the ideal treatment duration, dosing spacing methods and long-term cost-effectiveness.

### Clinical implications

These results provide clinical support for a customized biologic selection strategy, though choices must be tempered by the fact that many confidence intervals overlap substantially between the top-ranked biologic regimens, meaning small ranking differences should not be over-interpreted. Patients who prioritize rapid symptom alleviation and olfactory recovery remain highly suited for dupilumab. (Lane et al., 2025; Whitcroft et al., 2023). Patients with a high polyp burden may potentially benefit from benralizumab given its favorable ranking for NPS, although this finding should be interpreted cautiously because of overall network inconsistency, moderate heterogeneity, and the limited availability of direct comparative evidence (Han et al., 2021; Wang et al., 2025), Omalizumab continues to offer distinct advantages in patients with comorbid allergic disease, while mepolizumab may be better suited for patients with eosinophilic CRSwNP and asthma overlap (Bachert et al., 2022b; Papacharalampous et al., 2024). These results emphasize the role of precision medicine and endotyping in guiding treatment decisions, while acknowledging the overlapping therapeutic boundaries highlighted by the network structure (Toppila-Salmi et al., 2025). Cost considerations remain important, and recent cost-effectiveness analyses highlight the need for judicious use of biologics within healthcare systems (Yong et al., 2023). Although stratified analyses by inflammatory endotype or comorbidities were not feasible due to inconsistent reporting across trials, future trials that report results by endotype will be crucial to provide more accurate comparison analyses and allow customized biologic selection. In order to match therapies with patient-specific disease profiles and objectives, integrating biologics into CRSwNP care ultimately necessitates coordination across specialties, including otolaryngology, allergies, and pulmonology.

### Limitations

This study has several limitations. Although our network meta-analysis included 16 randomized controlled trials and more than 4,800 patients, the number of direct head-to-head comparisons among biologics was limited. The findings should also be interpreted considering the assumptions of network meta-analysis. Indirect comparisons rely on the assumption of transitivity across trials, and potential differences in study populations, dosing regimens, or treatment duration may introduce residual heterogeneity. The NPS outcome showed moderate heterogeneity, which is probably due to differences in trial design, dosage schedules, length of treatment, and patient comorbidities, such as asthma or NSAID-exacerbated respiratory disease, which are known to affect disease severity and response. Additionally, the SNOT-22 network demonstrated substantial heterogeneity and significant inconsistency, and the NPS network exhibited significant inconsistency as well. These findings reduce confidence in some indirect treatment comparisons and highlight the limitations inherent to network meta-analysis when direct head-to-head evidence is limited.

Although the robustness of our results was supported by sensitivity analyses that excluded high-risk-of-bias studies, internal validity may still be affected by issues such as missing outcome data and selective reporting in a subset of trials. Furthermore, most studies examined outcomes over 24–52 weeks, raising questions about the long-term durability and safety of these treatments. Furthermore, while standardized mean differences allow for cross-study pooling, their interpretation may be more difficult than raw clinical units. Finally, stratifying biologic therapy by dose and frequency reduced the amount of data available for individual comparisons, thereby decreasing the confidence of some estimates compared to analyses that pool treatments at the drug level. Publication bias and small-study effects cannot be entirely ruled out, particularly for SNOT-22 and UPSIT outcomes.

Longer-term results, dose-ranging trials, and direct head-to-head comparisons of biologics should be the main topics of future study. Phenotype-driven treatment strategies would be validated by trials stratified by patient endotype (allergic, eosinophilic, mixed).

## Conclusion

Our network meta-analysis shows that biologic treatments significantly improve outcomes in CRSwNP. Dupilumab ranked highest for improving quality of life (SNOT-22) and olfactory function (UPSIT), whereas benralizumab ranked highest for reducing polyp load (NPS). Omalizumab and mepolizumab also provided clinically significant advantages, though to a lesser extent. These findings highlight biologics as a revolutionary therapy option for individuals with severe, refractory CRSwNP. However, heterogeneity and inconsistency observed in the SNOT-22 and NPS networks warrant cautious interpretation of treatment rankings.

## Data Availability

The original contributions presented in the study are included in the article/Supplementary Material, further inquiries can be directed to the corresponding author.
