# Supplementary material for: Impact of dose and administration schedule on biologic efficacy in chronic rhinosinusitis with nasal polyps: a network meta-analysis
Source: Front Pharmacol. 2026 Aug 7;17:1772373. doi: 10.3389/fphar.2026.1772373 (PMC13494997; doi:10.3389/fphar.2026.1772373)
Supplement: Supplementary file 1 [file Supplementaryfile1.docx]

**Figure S1.** Node-splitting analysis of local inconsistency for Nasal Polyp Score (NPS) network meta-analysis.

**Figure S2.** Node-splitting analysis of local inconsistency for Sino-Nasal Outcome Test-22 (SNOT-22) network meta-analysis.

**Figure S3.** Node-splitting analysis of local inconsistency for University of Pennsylvania Smell Identification Test (UPSIT) network meta-analysis.
